# Activation function 1 of progesterone receptor is required for mammary development and regulation of RANKL during pregnancy

**DOI:** 10.1038/s41598-022-16289-x

**Published:** 2022-07-19

**Authors:** Shi Hao Lee, Yeannie H. Y. Yap, Chew Leng Lim, Amanda Rui En Woo, Valerie C. L. Lin

**Affiliations:** 1 School of Biological Sciences, Nanyang Technological University, Singapore, 637551 Singapore; 2 Department of Oral Biology and Biomedical Sciences, Faculty of Dentistry, MAHSA University, Bandar Saujana Putra, 42610 Jenjarom, Selangor Malaysia

**Keywords:** Developmental biology, Molecular biology

## Abstract

Progesterone receptor (PGR) is a member of the nuclear receptor superfamily of transcription factors. It is critical for mammary stem cells expansion, mammary ductal branching and alveologenesis. The transcriptional activity of PGR is mainly mediated by activation functions AF1 and AF2. Although the discovery of AF1 and AF2 propelled the understanding of the mechanism of gene regulation by nuclear receptors, their physiological roles are still poorly understood. This is largely due to the lack of suitable genetic models. The present study reports gain or loss of AF1 function mutant mouse models in the study of mammary development. The gain of function mutant AF1_QQQ exhibits hyperactivity while the loss of function mutant AF1_FFF shows hypoactivity on mammary development. However, the involvement of AF1 is context dependent. Whereas the AF1_FFF mutation causes significant impairment in mammary development during pregnancy or in response to estrogen and progesterone, it has no effect on mammary development in nulliparous mice. Furthermore, *Rankl,* but not *Wnt4* and *Areg* is a major target gene of AF1*.* In conclusion, PGR AF1 is a pivotal ligand-dependent activation domain critical for mammary development during pregnancy and it exerts gene specific effect on PGR regulated genes.

## Introduction

The mammary gland is an exocrine gland that provides milk for the young. Its development occurs mainly in the adult in response to ovarian steroid hormones estrogen and progesterone. It consists of highly branched mammary ducts and alveoli that are organized in lobules by the surrounding stromal tissue. Mammary ducts and alveoli are lined by the inner luminal epithelia and outer myoepithelia (often called the basal cells). The mammary stem cells (MaSCs) are defined by the expression of specific surface markers and by their ability to regenerate a functional mammary gland in cleared mammary fat pad transplantation. Earlier studies showed that MaSCs are enriched in CD45^−^Ter119^−^CD31^−^CD29^hi^CD24^+^ or CD45^−^Ter119^−^CD31^−^CD49f^hi^CD24^+^ population^1,2^. It is now known that there are distinct lineages of MaSCs (or progenitors) based on stages of mammary development, cell fate and the expression of estrogen receptor (ER) and progesterone receptor (PGR) (see Reviews in^3,4^). Cells giving rise to lineage-specific progeny are defined as progenitor cells^4^. Basal progenitor cells give rise to ductal and alveolar myoepithelial cells, whereas the luminal progenitors gives rise to ER^−^PGR^−^ and ER^+^PGR^+^ progenitor cells that, in turn, generate the ER^−^PGR^−^ ductal and alveolar luminal cells and ER^+^PGR^+^ ductal luminal cells, respectively^5,6^. The findings underscore the importance of ER and PGR signaling in mammary gland development.

Progesterone is essential for mammary morphogenesis. Classical experiments of ovariectomy and hormone replacement showed that estrogen was required for mammary ductal growth, whereas progesterone together with estrogen were necessary for the development of mammary alveoli^7,8^. The activity of progesterone is mediated by the PGR, which exists as isoform A and isoform B that are transcribed from two distinct promoters of Pgr gene^9^. Female mice with *Pgr* gene knockout exhibits lack of mammary ductal branching during pubertal development and paucity of alveologenesis during pregnancy^10^. The requirement of epithelial PGR for mammary morphogenesis was established in mammary epithelium transplantation experiment, in which cleared mammary fat pad transplanted with PGR (−/−) epithelium showed neither side branching nor alveologenesis during pregnancy^11^. Selective ablation of PGR isoform B (PGRB) expression in mice resulted in severely impaired side branching and alveolar development in response to progesterone and during pregnancy^12^. On the other hand, mice with PGR isoform A (PGRA) ablation showed similar mammary development as the wild type (WT) mice. This suggests that PGRB plays a major role in mediating progesterone induced mammary ductal side branching and alveologenesis.

PGR is a member of nuclear receptor superfamily that are transcription factors. Gene expression profiling revealed hundreds of progesterone regulated genes in the mammary tissue^13^. A number of progesterone regulated genes have been studied for their involvement in mammary development. These include cyclin D1^14,15^, Receptor Activator of Nuclear factor Kappa-Β Ligand (RANKL)^16,17^, WNT4^18,19^, RSPO1^20^, AREG^21^ and ADAMTS18, etc.^22^. These proteins can mediate PGR action by intrinsic or paracrine signalling. For example, progesterone can directly stimulate mammary epithelial proliferation in PGR-positive cells through increasing the activity of cyclin D1; progesterone can also promote PGR-negative mammary epithelial cell proliferation through RANKL released from PGR-positive cells^23^. Ablation of RANKL abolished progesterone-induced mammary growth, and RANK overexpression in MMTV-RANK transgenic mice led to accelerated mammary growth and tumorigenesis^23,24^. WNT4 is required for mammary ductal side-branching early in pregnancy when progesterone stimulates *Wnt4* expression^18^. AREG, an EGFR ligand, is also a major paracrine factor for estrogen induced ductal growth^25^. Estrogen-induced AREG in ER-positive luminal cells exerts paracrine effect by inducing the expression of *Rspo1* in ER-negative luminal cells, which signal through WNT pathway for mammary development^26^. Progesterone also induces the expression of mammary *Areg* during puberty to promote terminal end bud formation^21^.

Progesterone is a major hormonal factor for promoting MaSC expansion in adult mice^27^. RANK, RANKL, WNT4 and RSPO1 are critical mediators of progesterone-induced MaSC expansion^27,28^. *Wnt4* expression in adult mice was found to be specific to PGR-positive luminal cells and the secreted WNT4 signals to basal cells for MaSC expansion. It was also reported that progesterone-induced WNT4 in PGR^+^ luminal cells and RSPO1 in PR^−^ luminal cells work together to maintain MaSC niche, while RSPO1 signalling also upregulates ESR1 independent of WNT4^19,20,29^. On the other hand, WNT4 promotes the expression of *Adamts18* in myoepithelial cells, which in turn is important for maintaining the MaSC pool^22^. It is also reported that RANK/RANKL signalling strengthens the effect of WNT4 on progenitor cells for MaSC expansion^28^. It is thus conceivable that these various mediators of progesterone/PGR act in synergy in a cellular and developmental context specific manner to bring about homeostatic mammary growth and maintenance.

The transcriptional activity of nuclear receptor in general is primarily mediated by activation functions (AF), AF1 and AF2. AF1 is in the intrinsically disordered N terminal domain (NTD), whereas AF2 is in the structurally conserved ligand binding domain (LBD). AF1 was first discovered as a ligand-independent activation domain whereas AF2 was found to mediate the ligand-dependent response^30^. AF1 and AF2 mediate the recruitment of transcription coregulators for chromatin modelling and assembly of general transcription machinery^31,32^. It is widely believed that AF1 plays critical roles in gene and cellular context-dependent function of PGR^32^. This is explained by its intrinsically unstructured nature that acquires structures upon interaction with context specific partner proteins^33^, which in turn offer functional versatility. Studies of mouse models with ERα AF1 or AF2 domain deletion showed tissue-specific involvement of AF1 in several physiological processes. For example, AF1 is required for the development of trabecular bone and uterus whereas AF2 is necessary for cortical bone growth^34,35^. In the mammary gland, both AF1 and AF2 are required for luminal cells with high levels of ERα to induce *Areg, Wnt4* and *Pgr1*, whereas AF2 is sufficient for regulation of genes for cell adhesion and cytoskeletons in cells with low levels of ERα^36^. However, no other nuclear receptors have been studied for the physiological functions of AF1 and AF2.

In order to understand PGR AF1 function, we identified three critical amino acids (K464, K481, R492) of AF1 that are monomethylated in human PGR^37,38^. The hypomethylation mimic AF1_QQQ mutant (K464Q_K481Q and_R492Q) was hyperactive, whereas the hypermethylation mimic AF1_FFF mutant (K464F_K481F_R492F) was hypoactive in gene reporter assays^38^. The three residues are evolutionally conserved and correspond to K461, K478 and R489 in the mouse. AF1_QQQ (K461Q_K478Q_R489Q) and AF1_FFF mouse lines (K461F_K478F_R489F) were generated by CRISPR-Cas9 mediated gene editing. Consistent with the in vitro activities, the AF1_QQQ mutant exhibited greater levels of mammary morphogenesis and the AF1_FFF mutation showed less as compared to the WT mice. The study also indicated that AF1 activity is important for MaSC expansion, and RANKL is a key mediator of AF1 function.

## Results

### The AF1_QQQ mutant is more active in promoting mammary development

**Figure 1:**
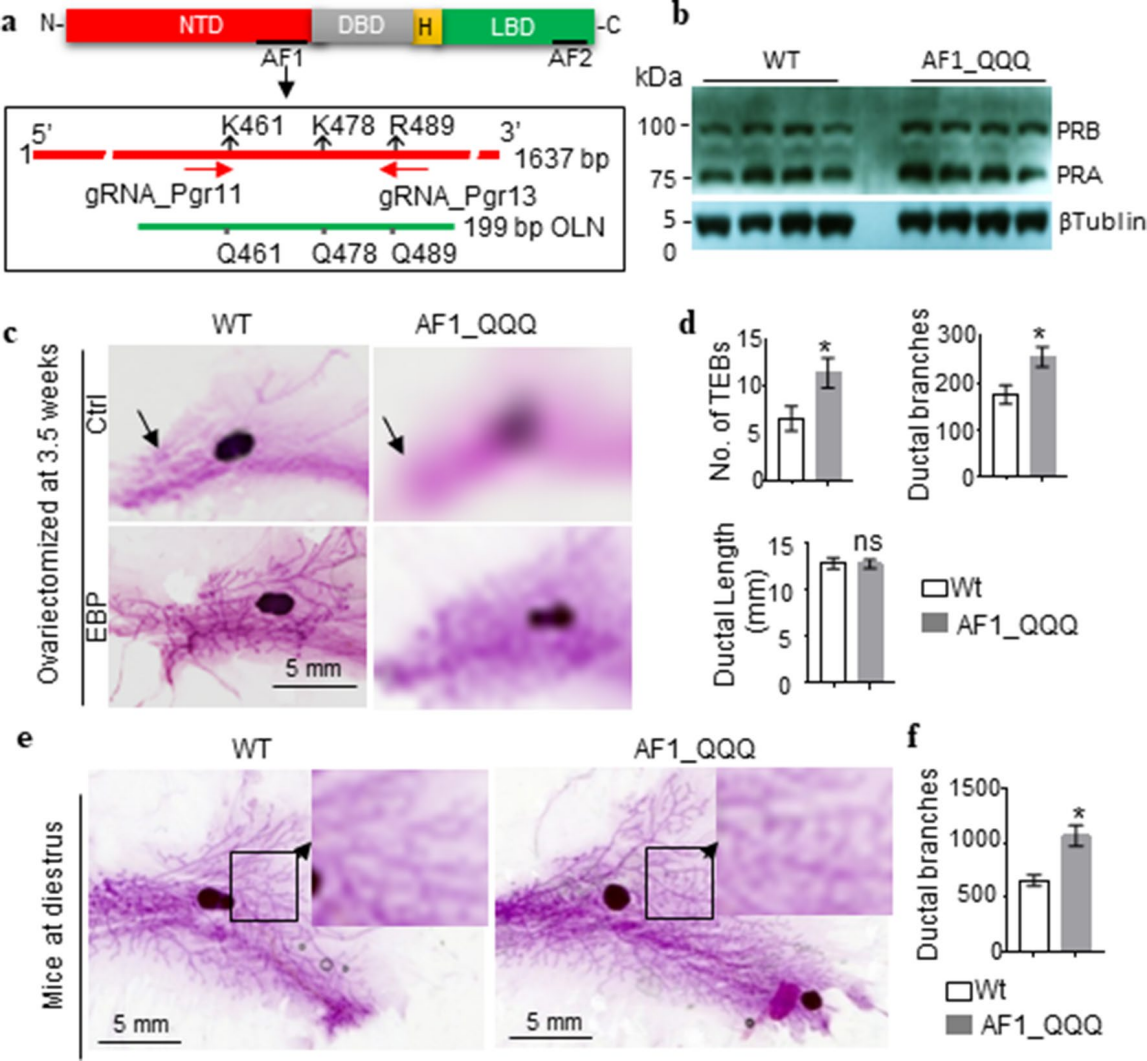
AF1_QQQ mutations promote mammary development in response to estrogen and progesterone. (**a**) Domain structure of PGR and CRISPR-Cas9 gene editing strategy. The red bar indicates exon 1 of *Pgr* gene (1–1671 bp). Cas9 mRNA, 2 *Pgr* gRNAs and the homologous single-stranded oligonucleotide (OLN) containing the 3 mutations were injected into the pronuclei for mutant generation. (**b**) AF1_QQQ mutations did not significantly affected PGR levels in estrogen-treated ovariectomized mice. (**c**,**d**) AF1_QQQ mice show greater number of TEB and ductal branching in response to EBP. Mice were ovariectomized at 3.5 weeks old. After 2 weeks, the mice were treated with control vehicle (Ctrl) or EBP for 16 days. (**c**) Representative whole mount images of mammary gland. Rudimentary mammary ducts in Ctrl are indicated by arrows. (**d**) Quantification of TEBs, mammary ductal branches and ductal length from the whole mount images of EBP-treated mammary gland (mean ± SEM, *p < 0.05, n = 6). The vehicle-treated mammary gland only contains a few rudimentary ducts and were therefore not quantified. (**e**,**f**) AF1_QQQ mice at dioestrus also show greater number of mammary ductal branching than the WT mice. (**e**) Representative whole mount mammary images with enlarged insets to show greater details. (**f**) Quantification of ductal branching (results are expressed as mean ± SEM, n = 6, *p < 0.05).

The mouse PGR lacks the amino acid proline-leucine-proline (PLP) corresponding to amino acids 425–427 in human PGR AF1. Hence, K461, K478 and R489 in mouse PGR correspond to K464, K481 and R492 in human PGR, respectively (Fig. 1a). With the consideration that the hypoactive AF1_FFF mutant female mouse would be infertile due to the lack of PGR activity, we initially chose to generate the hyperactive AF1_QQQ mutant by CRISPR-Cas mediated gene editing to evaluate AF1 function (Fig. 1a). Both the heterozygous and homozygous AF1_ QQQ mice appear healthy and fertile. Heterozygous breeding produced offspring with homozygous, heterozygous, and WT genotypes according to the Mendelian inheritance ratio of 1:2:1.

The effect of AF1_QQQ mutations on mammary development was investigated in mice ovariectomized (OVX) at 3.5 weeks old. Following a daily injection of 17β-estradiol benzoate (EB) and progesterone (EBP) for 16 days, the levels of PGR in WT and AF1_QQQ mice are similar (Fig. 1b; the unprocessed gel images are shown in Supplementary Fig. S1). Whole mount analysis of the 4th mammary gland (MG) from the vehicle control groups showed rudimentary mammary development with no significant difference between genotypes (Fig. 1c). This is expected because mammary growth hardly occurs in the absence of ovarian steroid hormone. EBP treatment in the WT mice stimulated mammary development and mammary ducts extend well beyond the lymph node. In contrast, the mammary development in EBP-treated AF1_QQQ mice is more extensive than the WT mice (Fig. 1c). The number of mammary terminal end buds (TEBs) with distinct bulbous feature and mammary ductal branching points in AF1_QQQ mice were significantly more than the WT (Fig. 1d). However, the ductal lengths were similar between the genotypes. The observations suggest that AF1 is important for progesterone stimulation of mammary TEB growth and ductal side branching.

We also evaluated the effect of AF1_QQQ mutation on mammary development in intact mice under physiological conditions. In mice, the circulating estrogen rises in the pro-estrus and estrus, whereas the level of progesterone starts to rise at later phase of metestrus and reaches a peak at dioestrus. AF1_QQQ mice of 9–10 weeks old at dioestrus displayed a more pronounced lobuloalveolar structures and tertiary ductal branching (*p* < 0.05) in comparison to WT mice (Fig. 1e,f). It is to note that the larger number of branching points in AF1_QQQ mice owes to the alveolar structures that develop at the ends of the tertiary branches. There were only few TEBs that fulfil the criteria of > 0.012 mm^2^ at this stage of the development and it is not feasible to quantify the difference of TEB between the genotypes.

Since progesterone is important for the expansion of adult MaSC in mice, we evaluated whether the AF1_QQQ mutations affected MaSC expansion using mice at dioestrus. The 4^th^ MG was analyzed by FACS for CD45^−^Ter119^−^CD31^−^ (Lin^−^) CD24^+^CD49f^hi^population that are enriched with MaSCs activity^1,2^ (Supplementary Fig. S2a). The percentage of Lin^−^CD24^+^CD49f^hi^ cells relative to Lin^−^ cell population in AF1_QQQ MG was higher than that in the WT mice but the difference was statistically not significant (p > 0.05) (Supplementary Fig. S2b). It is to be noted that when the percentage of Lin^−^CD24^+^CD49f^hi^ cells is significantly higher when it is expressed relative to live cell population (p < 0.05). This suggests that AF1 is potentially involved in progesterone regulation of MaSCs.

### The AF1_FFF mutant is hypoactive in promoting mammary development

To gain a more in depth understanding of the function of AF1, we generated AF1_FFF mutant mice using the same gene editing strategy as that for AF1_QQQ except that the homologous OLN contains the FFF mutations. The homozygous FFF mutant mice are 100% infertile.

**Figure 2:**
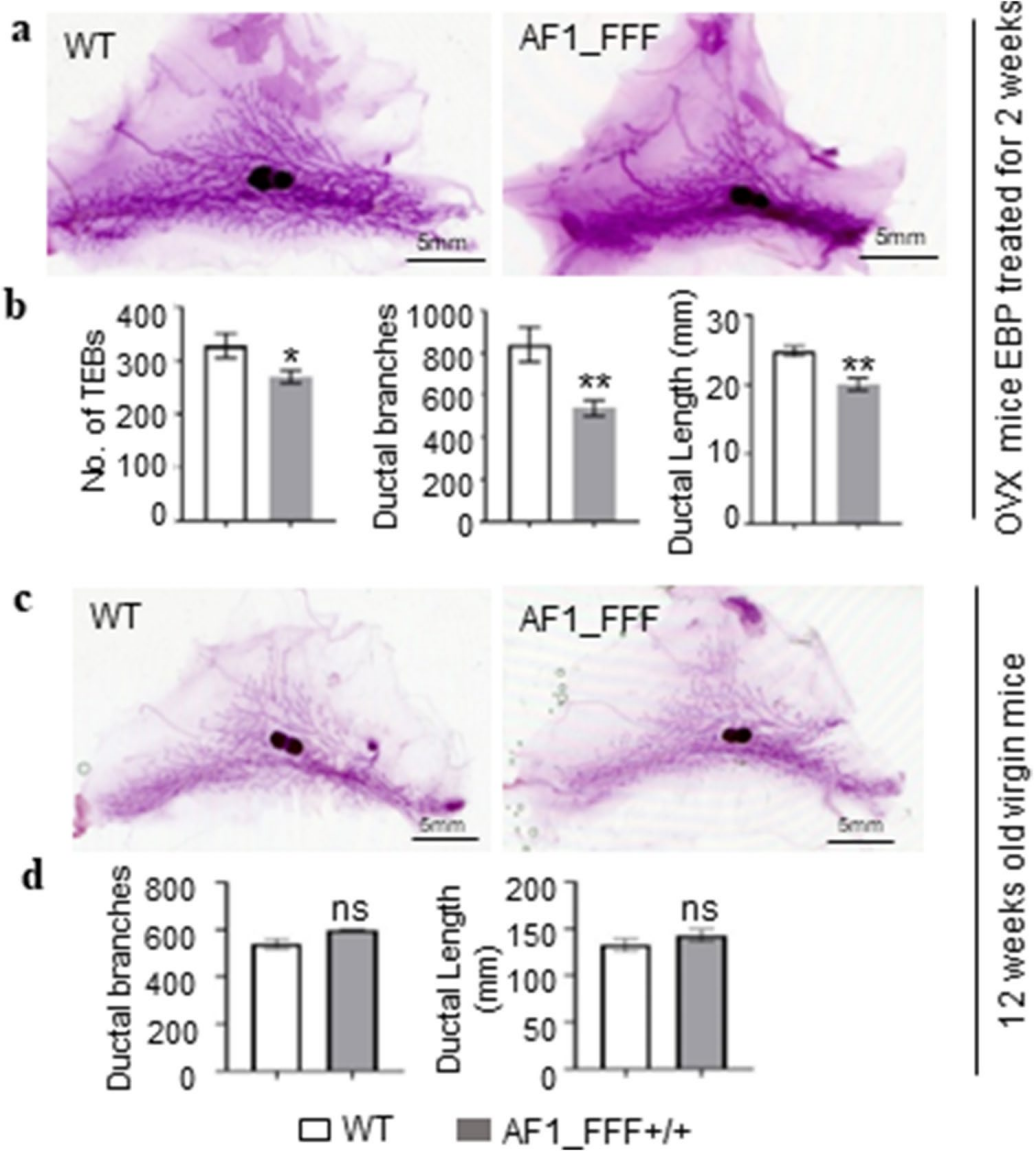
AF1_FFF mutations result in reduced mammary development in response to EBP but have no effect on intact nulliparous mice. (**a**,**b**) Mice ovariectomizd at 6 weeks old were treated with EBP for two weeks before whole mount analysis of the 4th mammary gland. (**a**) Representative whole mount images; (**b**) quantification of the TEB, ductal branching and ductal length by image analysis (n = 5 for WT and n = 6 for AF1_FFF). (**c**,**d**) No difference in mammary development between the WT and AF_FFF mutant virgin mice at 12 weeks old. (**c**) Representative whole mount images; (**d**) quantification of the ductal branching and length (n = 4 for each genotype). All numeric data are expressed as mean ± SEM, *p < 0.05; **p < 0.01, *ns* not significant.

We initially investigated the effect of AF1_FFF mutations on MG development in 6 weeks old OVX mice given daily EBP treatment for 10 days. The wholemount image analysis showed clear impairment in mammary development in AF1_FFF mice (Fig. 2a). There were significantly less TEBs (p < 0.05), less mammary side branches (p < 0.01) and shorter ductal length (p < 0.01) in AF1_FFF mice compared to the WT mice (Fig. 2b). This suggests that AF1_FFF mutations causes loss of function and AF1 is critical for progesterone to promotes mammary development.

We further assessed the effect of AF1_FFF mutation in intact nulliparous mice without synchronizing the estrous cycle. The mammary gland in 12 weeks old mice of both genotypes have extensive ductal growth, but there were no notable TEBs that meet criteria of ≥ 0.012 mm^2^ in area. There were neither differences in mammary ductal branching nor ductal length between the genotypes (Fig. 2c,d). We also kept nulliparous mice till 14–16 months old and found no difference in mammary development between the WT and the AF1_FFF mice (Supplementary Fig. S3). Together, these data suggest that PGR AF1 is required for mammary development in the presence of high levels of estrogen and progesterone but not necessary for mammary development in nulliparous mice under physiological conditions.

### Pregnant AF1_FFF mammary gland shows reduced MaSC

**Figure 3:**
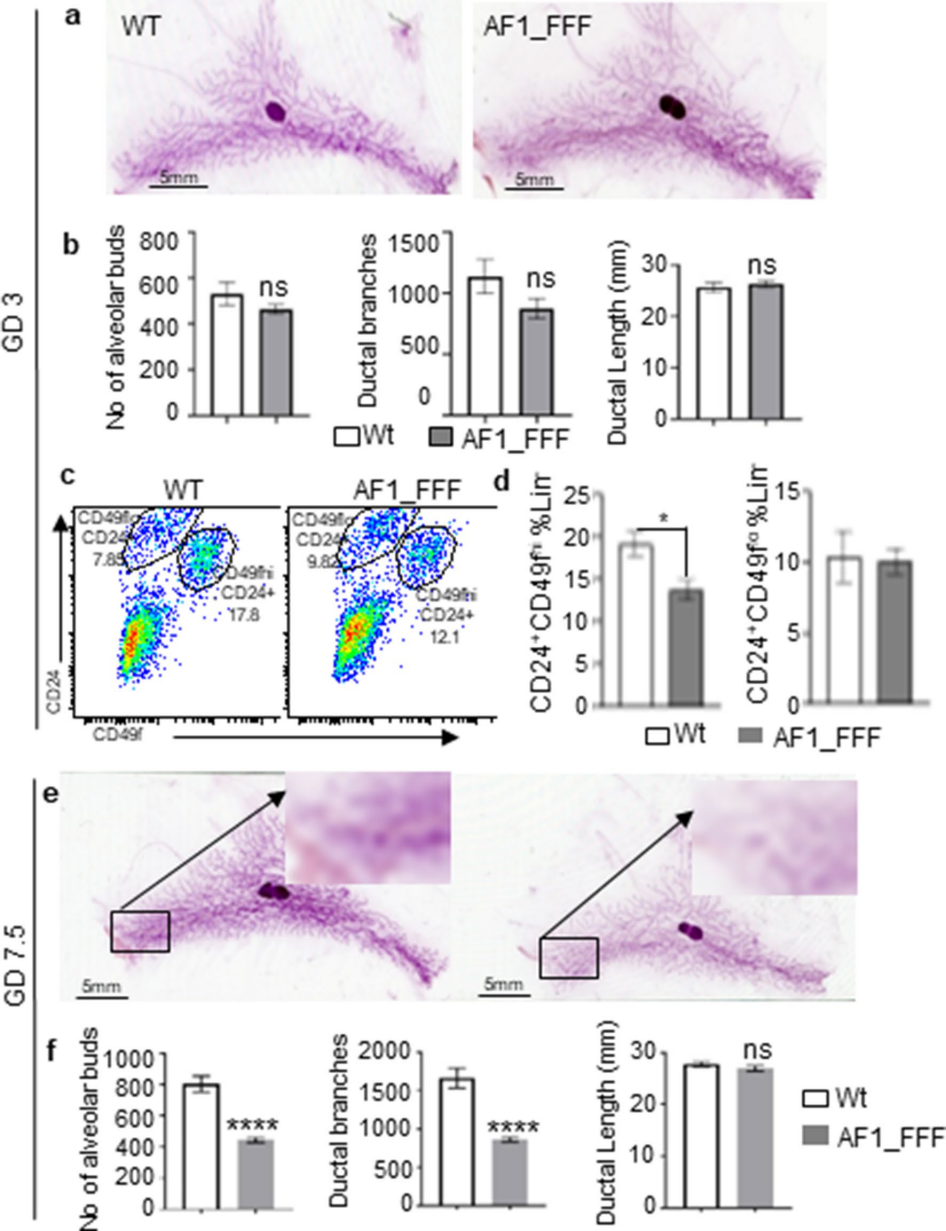
AF1_FFF mutations lead to reduced mammary growth during pregnancy. (**a**,**b**) Mammary development on GD 3 is similar between the WT and AF1_FFF mice. (**a**) Representative whole mount images on GD3; (**b**) quantitative data of alveolar buds, ductal branching and ductal length (n = 7 for WT, n = 8 for AF1_FFF, *ns*, not significant). (**c**,**d**) Significant less MaSC (Lin^−^CD49f^hi^CD24^+^) in AF1_FFF mice on GD3 (*p < 0.05). (**c**) FACS dot blots showing Lin^−^CD24^+^CD49f^lo^ and Lin^−^ CD24^+^CD49f^hi^ cell gating. (**d**) Percentage of Lin^−^CD24^+^CD49f^lo^ and Lin^−^CD24^+^CD49f^hi^ cells relative to Lin^−^ cell populations (WT, n = 4; AF1_FFF, n = 4, *p < 0.05). (**e**,**f**) Mammary development on GD7.5 in AF1_FFF mice is significantly impaired. (**e**) Representative whole mount images; (**f**) quantitative data of the alveolar buds, ductal branching and ductal length (WT, n = 10; AF1_FFF, n = 11, ****p < 0.001). All numeric data are expressed as mean ± SEM.

To verify whether AF1 is necessary for mammary development and MaSC expansion in the presence of high levels of estrogen and progesterone, we evaluated MG development in the first week of pregnancy. There is a ninefold increase (from 4.9 to 45.5 ng/ml) in the circulating levels of progesterone from Gestation Day 1 (GD1) to GD3, and the high level of progesterone were maintained in the first week^39^. Although the AF1_FFF mice are infertile, some of the mice mated at 12–13 weeks old could get conceived and the foetus persisted till mid pregnancy albeit severely developmentally impaired. The whole mount images from pregnant mice showed bulbous ends structures akin to the alveolar structures at the ends of the tertiary branches. Since TEBs are conventional features for pubertal mammary development, we refer these structures during pregnancy as alveolar buds. Consistent with the observation in 12–13 weeks old nulliparous mice, the mammary gland at GD3 showed no significant difference in the number of alveolar buds and side ductal branching between the WT and AF1_FFF mice (Fig. 3a,b). However, there were significantly lower percentage of Lin^−^CD24^+^CD49f^hi^ basal cells in the AF1_FFF mutant (Fig. 3c,d), suggesting that AF1 is required for progesterone stimulation of MaSC expansion under physiological conditions. At GD7.5, mice showed greater mammary development compared with that at GD3, and the AF1_FFF mice had significantly less mammary alveolar buds and ductal branches (p < 0.0001) than the WT mice (Fig. 3e,f). The observations indicate that PGR AF1 is involved in mediating progesterone induction of MaSC expansion and MG development.

### The expression of *Rankl* was significantly reduced in AF1_FFF mammary tissue

**Figure 4:**
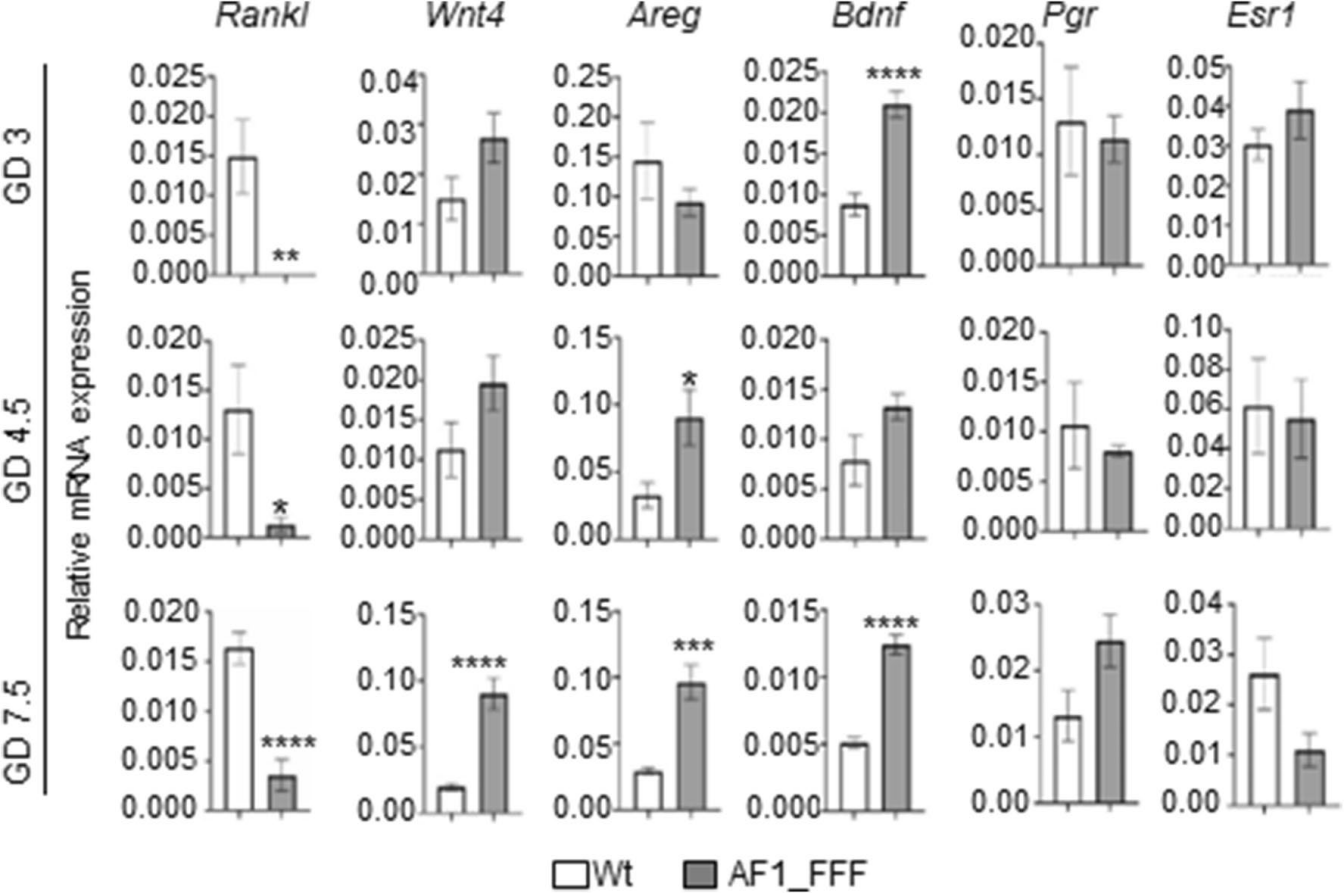
Effect of AF1_FFF mutations on the expression of Pgr and PGR target genes important for MG development. Total RNA from the mammary gland on GD3, GD4.5 and GD7.5 were analyzed for gene expression by RT-qPCR using 36B4 primers as internal control. The results are expressed relative to 36B4 gene (mean ± SEM, n = 6 for all groups). *Denotes statistically significant difference between the WT and the mutant.

To understand the molecular mechanism by which AF1 mediates progesterone regulation of mammary development, we tested the expression of several well characterized paracrine factors that are downstream PGR signalling^40^. The TNF family protein, receptor activator of NFκB ligand (RANKL) is a key promoter of mammary morphogenesis and MaSC expansion^27,41^. The expression of *Rankl* was significantly downregulated in the AF1_FFF mammary tissue on GD3, GD4.5 and GD7.5 (Fig. 4). WNT4 is a family of WNT proteins that signal to Frizzled family of G protein coupled receptors. It is induced by progesterone in the mammary gland and is required for mammary development^18^. Surprisingly, *Wnt4* gene expression was increased in the mutant at all three time points although the increase was only statistically significant on GD7.5 (Fig. 4, p < 0.0001). Similarly, the expression of amphiregulin (*Areg*) and brain-derived neurotrophic factor (*Bdnf*), both of which are important progesterone regulated genes for mammary cell growth^21,42,43^, were also significantly upregulated in the AF1_FFF mammary tissue in early gestation (Fig. 4). However, AF1_FFF mutations had no significant effect on the expression of *Pgr* or *Esr1* although *Pgr* expression on GD7.5 showed a trend of upregulation (p > 0.05).

### RANKL protein is markedly downregulated in AF1_FFF mice at early pregnancy.

**Figure 5:**
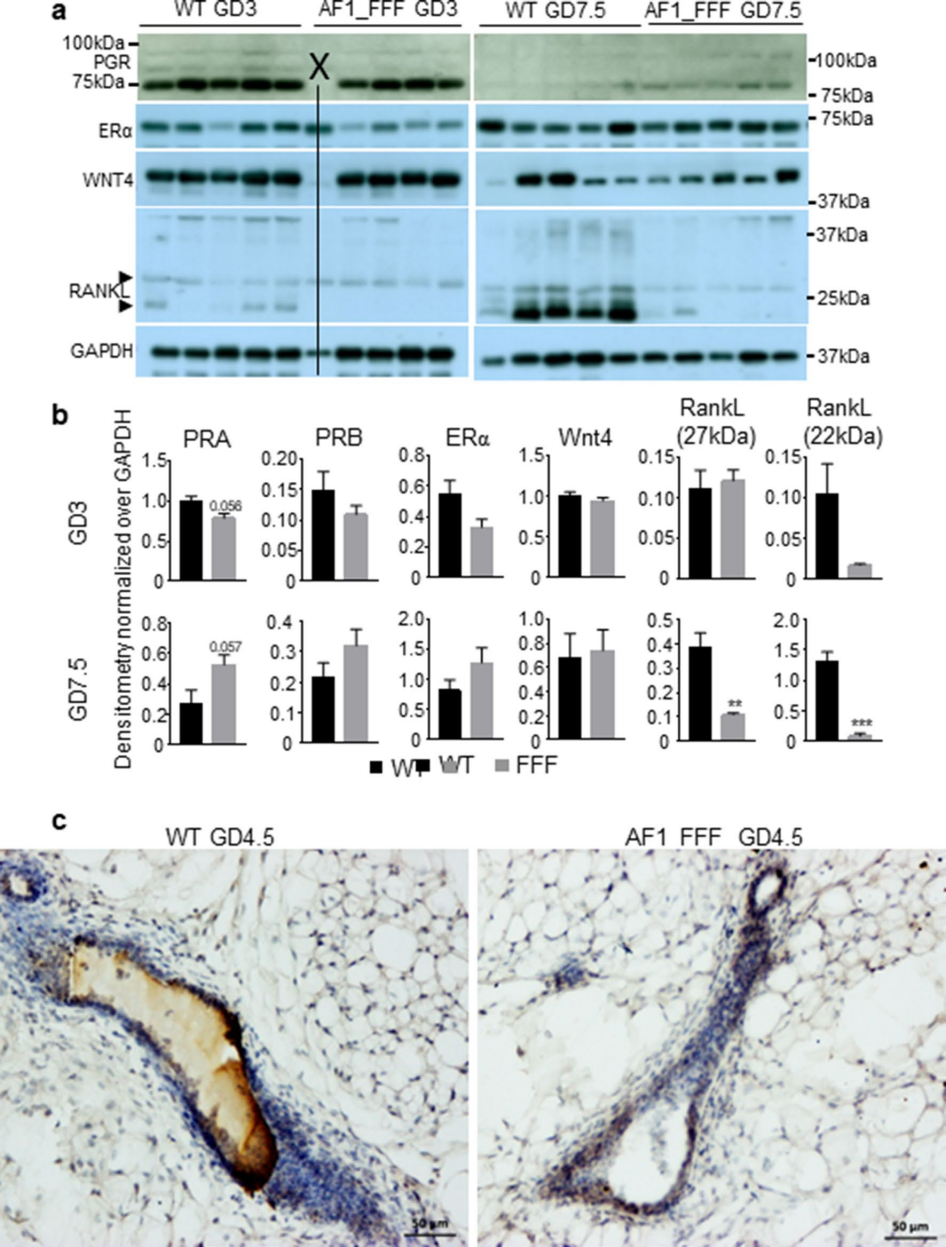
RANKL protein was significantly reduced in AF1_FFF mammary tissues in early pregnancy. (**a**) Protein levels of PR, ERα, WNT4 and RANKL in the mammary tissue were analysed by Western blotting analysis. Lanes indicated by X followed by a line is a defective sample. There is a upshift of PR protein bands on GD7.5 as compared to that on GD3. The major RANKL protein bands were detected are ~ 27 kDa and 22 kDa (indicated by black arrow heads) which correspond to soluble RANKL proteins. The level of GAPDH was used as a loading control. (**b**) Quantification of protein levels by densitometry. The results are expressed as mean ± SEM. **p < 0.01; ***p < 0.001. (**c**) Representative images of WT and AF1_FFF mammary sections probed with RANKL antibody and detected using VECTASTAIN Elite ABC HRP Kit.

Western blotting analysis was conducted to determine the protein levels of PGR, ERα WNT4 and RANKL in mammary tissue on GD3 and GD7.5. The unprocessed gel images are shown in Supplementary Fig. S4. There are several interesting observations on PGR blots (Fig. 5a). First, PGRA is the major PGR protein (~ 7 times more than PGRB) on GD3 but the level drops markedly on GD7.5 such that the PGRA level was only slightly higher than PGRB on GD7.5. Progressive decrease of PGRA levels during pregnancy was also reported by Aupperlee et al.^44^. Second, PGR bands are visibly upshifted on GD7.5 compared to GD3. The molecular mass of mouse PGRA and PGRB based on amino acid composition are 81.75 kDa and 98.98 kDa, respectively. On GD3, PGRA appears at ~ 75 kDa and a faint band below 100 kDa that is assumed to be PGRB. On GD7.5, the PGRA band is well above the 75 kDa and the PGRB is above 100 kDa. It is likely that PGR undergoes posttranslational modifications to regulate PGR activity depending on the stages of mammary development. Third, the levels of PGR proteins appear lower in the mutant on GD3 but higher on GD7.5 in the mutant, although the changes are marginally significant for PGRA (Fig. 5b, p = 0.06). On the other hand, ERα levels were not affected by AF1_FFF mutations. There was no difference in WNT4 proteins levels between the genotypes on GD3 and GD7.5 although the mRNA level was reduced.

Consistent with the gene expression data, RANKL protein level was reduced in the mutant on GD3 and GD7.5. As is shown in Fig. 5a, the major RANKL protein bands detected are ~ 27 kDa and 22 kDa which correspond to soluble RANKL protein. The 22 kDa RANKL was reduced on GD3 with marginal significance (Fig. 5b, p = 0.09). Both 27 kDa and 22 kDa RANKL were significantly reduced on GD7.5. This is confirmed by immunohistochemistry (IHC) of MG sections with RANKL antibody, which showed a stronger brownish immune reactive RANKL in the lumen and in mammary epithelial cells of the WT section than the AF1_FFF section on GD4.5 (Fig. 5c). The RANKL IHC images from other mice are presented in Supplementary Fig. S5. Taken together, the study confirmed that the AF1_FFF mutation resulted in reduced levels of RANKL protein.

## Discussion

One of the landmarks in the research of nuclear receptors is the discovery of AF1 and AF2^30,31^. It opened the door for the understanding of how nuclear receptors regulate gene expression through the recruitment of transcription coregulators and chromatin remodelers. Despite the pivotal role of nuclear receptor in gene regulation in health and disease, the physiological significance of the individual activation function is poorly understood. The present study reports the first gain and loss of PGR AF1 function mouse models. Consistent with their in vitro activities, the AF1_QQQ mutant showed hyper response to progesterone in stimulating mammary ductal side branching and TEBs whereas the AF1_FFF mutant showed hypo response. The study also suggests that AF1 is required for MaSC expansion. The effect is likely exerted through targeting RANKL signaling as the mRNA and protein levels of RANKL were markedly downregulated in AF1_FFF mutant. Since RANKL is an important mediator of progestin elicited mammary tumorigenesis^24,45,46^, the study suggests a potential role of AF1 in the development of mammary tumors.

CD24^+^CD49f^hi^ cells are basal cell population enriched with MaSCs based on colony forming cell (CFC) and cleared mammary fat transplantation assay^1,2^. This cell population is amplified at dioestrus, or in response to progesterone treatment^47^. The involvement of AF1 in progesterone induced MaSC activity is indicated by significant reduction of CD24^+^CD49f^hi^ population in the AF1_FFF mice. As the basal cells are ER^−^PGR^−^, the effect on CD24^+^CD49f^hi^ population is likely mediated via paracrine factors such as RANKL, which is also downregulated in the AF1_FFF mutant. RANKL also exerts other paracrine effect on the mammary gland. For example, RANKL promotes the expansion of ER^−^PGR^−^ luminal progenitors through induction of RSPO1 and activation of WNT signalling^28^. RANKL also mediates the effect of progesterone on luminal progenitor cells differentiation through induction of ELF5^17^. It is conceivable that RANKL downregulation in the AF1_FFF mutant would not only cause reduced activity of MaSCs, but also decrease luminal progenitors and their differentiation potential. However, the conclusion on the involvement of AF1 in MaSC expansion is limited by the lack of functional characterization using either cleared mammary fat pad transplantation assay or in vitro colony formation assay. Furthermore, the notion is dampened by the lack of significant increase of Lin^−^CD24^+^CD49f^hi^ in the hyperactive AF1_QQQ mutant mice in dioestrus. Intriguingly, the expression of other downstream mediators of PGR including *Wnt4*, *Areg* and *Bdnf* were upregulated in AF1_FFF mice in early pregnancy. There can be several explanations for this observation. First, this may be the result of compensatory responses to the impaired PGR signaling because the upregulation of these genes appears progressive from GD3.5 to GD7.5. Take *Wnt4* for example, *Pgr* deletion did not abrogate *Wnt4* expression in perinatal mice^19^. In fact, EGFP expression driven by Wnt4 promoter (*Wnt4::Cre*) in the same study appears higher in mice with either *Esr1* or *Pgr* gene deletion. This suggests that the lack of PGR activity triggers a compensatory upregulation of *Wnt4* expression. This is also logical considering the observation that the effect of Wnt4 in promoting mammary development can be independent of PGR^18,19^. The impaired mammary development in AF1_FFF mice may thus trigger an upregulation of Wnt4 expression. Second, *Wnt4 and Areg* are also target genes of estrogen in the mammary gland^19,25^. It is possible that the reduced PGR signaling causes estrogen to further upregulate these genes. This is further supported by the observation that PGR, a bona fide estrogen target, also showed a trend of upregulation at the mRNA and protein level in the AF1_FFF mutant. The third scenario may involve errors in feedback loops such that the reduction of a downstream molecule in the negative feedback system causes the upregulation of an upstream molecule. A gene expression profiling would provide a clearer picture of the gene regulation defect in AF1_FFF mutant at the global level.

Although AF1_FFF mutations reduced mammary development during pregnancy, it did not seem to affect that in nulliparous mice. This could be attributed to differences in levels of progesterone and/or PGR property between these two developmental stages. First, circulating levels of progesterone during pregnancy is higher. Progesterone levels in cycling mice fluctuate from ~ 2 to 18 ng/ml in dioestrus^48^. In contrast, progesterone levels rise to 45 ng/ml from GD2 to GD3 and its level remains high throughout pregnancy^39^. Second, PGRA accounts for ~ 85% of the total MG PGR on GD3 but its level decreases sharply on GD7.5, which agrees with an earlier study that reports higher PRA to PRB ratio in nulliparous MG than pregnant MG^44^. It is conceivable that the reduction in the PGRA levels occur sometime after GD3 but the precise time is not clear. Third, both PGRA and PGRB undergo post translational modifications (PTM) during pregnancy as evidenced by an upshift of the PGR bands on GD7.5 compared to GD3 (Fig. 5). Protein phosphorylation is the primary cause for band upshift in Western blot and the vast majority of PGR phosphorylation occurs in the intrinsically disordered N terminal domain including AF1^49^. Taken together, it is tempting to speculate that the sustained high levels of progesterone enhance AF1 activity through PTM for more advanced mammary development. This is supported by the observation that AF1_FFF mice showed impaired MG development in response to estrogen and progesterone compared to the WT mice.

Studies of ERα suggested that the functions of AF1 and AF2 functions are linked to its expression levels and vary in different cell population^36^. Luminal mammary cells with high ERα expression require both AF1 and AF2 activities to induce transcription of important paracrine mediators such as *Areg, Wnt4* and *PGR* to induce ductal growth. It is conceivable that high level of estrogen is required to activate the high level of ERα in order to induce the activity of AF1 and AF2^36^. On the other hand, AF1 deletion had only subtle effect on cells with low ERα, suggesting cell context dependent activity of AF1. This raises an interesting possibility that PGR AF1 is only active in a subpopulation of epithelial cells under the influence of pregnancy-associated hormones and growth factors.

In conclusion, the study of mouse models with gain and loss of PGR AF1 function reveals critical roles of AF1 in the development of TEB and mammary ductal branching. The study also demonstrates that AF1 exerts gene-selective regulatory effect and RANKL is a key AF1 target gene, which may mediate AF1 regulation of basal MaSC expansion. However, AF1 is not necessary for MG development in intact nulliparous mice under physiological condition. It is possible that AF1 synergizes with AF2 in the cellular environment with high levels of estrogen and progesterone. In light of the significance of progesterone and RANKL signaling in MaSC expansion and mammary tumorigenesis, PGR AF1 is likely involved in mammary tumorigenesis.

## Materials and methods

### Ethical statement

All in vivo procedures and animal care performed are in accordance with the Institutional Animal Care and Use Committee (IACUC) guidelines set by National Advisory Committee for Laboratory Animal Research (NACLAR) of Singapore. The experiments were conducted based on approved protocol ARF SBS/NIE-A0304/A18004 by the Nanyang Technological University Institutional Animal Care and Use Committee (NTU-IACUC). All methods are reported in accordance with ARRIVE guidelines (https://arriveguidelines.org). Mice were housed under standard conditions under a 12 h dark/light cycle and been given ad libitum access to food and water.

### Generation of AF1 PGR mutant mice and genotyping

K461, K478 and R489 in the mouse PGR AF1 domain were mutated to glutamine (Q) or phenylalanine (F), respectively. PGR mutant mice were generated by CRISPR/Cas9 mediated nucleotide mutagenesis by the Animal Gene Editing Laboratory (AGEL), Biological Resource Centre of the Agency for Science, Technology and Research, Singapore. Two guide RNAs were designed to flanking the mutant region in the first exon of mouse PGR gene. The sequences are gRNA-PGR11: 5ʹ GGAGTGCATCCTGTACAAAG 3ʹ and gRNA-PGR13: 5ʹ GCGGCCGGCAGGCTGTCCCG 3ʹ (Fig. 1A). The mutation was introduced by single-stranded oligonucleotide (OLN) containing the respective triple mutations. gRNAs were synthesized by HiScribe™ T7 Quick High Yield RNA Synthesis Kit (NEB, #E2050), and Cas9 mRNA was synthesized by mMESSAGE mMACHINE T7 Ultra mRNA synthesis kit (Ambion, #AM1345). The pronuclei of one-cell embryos from C57BL/6 J were injected with a mixture of gRNAs (15 ng/ul each), Cas9 mRNA (25 ng/ul) and the template OLN (15 ng/ul) in 10 mM Tris.HCl, pH7.2 and implanted into pseudo-pregnant females. Founder animals were screened first by PCR with PGR1/PGR2 primers (5' AGCCAGCTCCTCCACCTTCCCAGAC 3' PGR2 (reverse primer): 5' AGGTAGTTAAGGTATGGCGGGTAGAC 3'), followed by Restriction Fragment Length Polymorphism (RFLP) of the PCR amplicons. The targeted mutations in the positive founders were confirmed by sequencing. Founder animals containing the desired mutation were bred with the wild type C57BL/6 J mice to produce F1 heterozygotes. The F1 mutants were identified by PCR and confirmed by sequencing.

The sequence of OLN for AF1_QQQ mutant (199nt) is the following: 5ʹAGCGCCGCGGTGTCGCCAGCGTCCTCCTCCGGCTCCGCGCTGGAGTGCATCCTGTACCAAGCGGAGGGCGCGCCGCCCACGCAGGGTTCGTTCGCGCCACTGCCGTGCCAGCCCCCAGCCGCCGGCTCCTGCCTACTACCCCAGGACAGCCTGCCGGCCGCCCCGGCCACCGCCGCAGCACCCGCCATCTACCAGCCGC 3ʹ.

The sequence of OLN for AF1_FFF mutant (199nt) is the following: 5ʹAGCGCCGCGGTGTCGCCAGCGTCCTCCTCCGGCTCCGCGCTGGAGTGCATCCTGTACTTTGCGGAGGGCGCGCCGCCCACGCAGGGTTCGTTCGCGCCACTGCCGTGCTTTCCCCCAGCCGCCGGCTCCTGCCTACTACCCTTCGACAGCCTGCCGGCCGCCCCGGCCACCGCCGCAGCACCCGCCATCTACCAGCCGC 3ʹ.

### Study of MG development in response to the estrogen and progesterone

Three weeks old female mice anesthetized with ketamine/xylazine anesthetic mixture were ovariectomized bilaterally and allowed to rest for 10–12 days post-surgery prior to hormone treatment. Animals were injected with either pure sesame oil as vehicle control group or with 17-β-estradiol benzoate (10 µg/kg) plus progesterone 10 mg/kg (EBP), subcutaneously. Injections were given continuously for the first two days and subsequently on alternate days for a period of 16 days (or 10 days for AF1_FFF mice). Treated mice were euthanized on the day after the last treatment.

### Study of MG development and MaSC expansion of AF1_QQQ mice at dioestrus

Nine to ten weeks old virgin female mice were examined and assigned to one of the four stages (proestrus, metestrus, estrus, and dioestrus) of the estrous cycle by vaginal smear cytology (Caligioni, 2009). Only mice that undergone normal estrous cycle transition for at least two cycles were included in this study. Mice at dioestrus were included in the study and vaginal smears were obtained again at the time of euthanization to confirm the animals’ dioestrus status.

### Whole mount and morphometric analysis of the mammary gland

Singly excised mice inguinal mammary glands (either fourth or ninth) were spread out onto glass slides, fixed with Carnoy fixative (6:3:1 mixture of ethanol:chloroform:glacial acetic acid) for at least four hours at RT, hydrated, and stained overnight in carmine alum solution (0.2% carmine and 0.5% aluminium potassium sulphate). The slides were dehydrated through a series of ethanol and cleared in Xylene thrice prior to mounting with DPX mounting media.

The distinct bulbous feature at the leading edge of advancing ducts were identified as TEBs^50^. TEBs, mammary ductal branching points, and ductal outgrowth length were determined from scanned mammary whole mount images (in 600 dpi) via HP Scanjet G4050. Only well-resolved TEBs with areas ≥ 0.012 mm^2^ were counted. Quantitative assessment of branching points was performed using MammoQuant^51^. Mammary ductal length was measured as the length from the primary duct to the leading edge of the duct at 10 × magnification.

### Mammary cell preparation and direct flow cytometry staining

Materials used for mouse mammary epithelial cells culture were purchased from STEMCELL Technologies (Vancouver, Canada). Single mammary cell suspension was generated from dissociated mouse mammary tissue according to manufacturer’s information sheet. In brief, resected inguinal mammary glands were dissociated overnight (~ 15 h) at 37 °C in complete EpiCult™-B Medium (Mouse) supplemented with 5% fetal bovine serum, 1 mg/ml collagenase, and 100 U/ml hyaluronidase. Upon dissociation, the resultant pellet was subjected to red blood cell lysis in ammonium chloride solution. To generate a single cell suspension of mammary epithelial cells, the partially dissociated tissue organoids were further digested with 0.25% trypsin–EDTA, 5 U/ml dispase, 1 mg/ml DNase I, and filtered through a sterile 30 µm nylon mesh. The cells in the filtrates were used for flow cytometry analysis.

All fluorochrome-conjugated mouse antibodies were purchased from Affymetrix eBioscience (San Diego, CA, USA) unless otherwise specified. Endothelial and haematopoietic cells were labelled with CD31-PE/Cy7 (390), and a combination of CD45-APC/Cy7 (30-F11) (Biolegend, San Diego, CA, USA) and Ter119-APC, respectively, to exclude the lineage-positive (Lin^+^) cells. Anti-CD49f FITC (eBioGoH3 (GoH3)) and Anti-CD24 PE (M1/69) were used to identify the mammary epithelial cell populations. Dead cells were excluded by irreversible labelling of cells with Fixable Viability Dye eFluor® 450. A total of live 50,000 cells were collected for each sample. Fluorescence of cell was measured with BD LSRFORTESSA™ X-20 and FlowJo V10 was used for subsequent data analysis.

### SDS-PAGE and western blotting

Snap frozen mammary glands were powdered in liquid nitrogen with a mortar and pestle. Total protein was extracted by homogenization in cold lysis buffer containing 50 mM HEPES, 150 mM sodium chloride, 1% Triton X-100, 5 μg/ml pepstatin A, 5 μg/ml leupeptin, 2 μg/ml aprotinin, 1 mM PMSF, 100 mM sodium fluoride, and 1 mM sodium vanadate (pH 7.5). Concentration of protein in tissue lysates was quantified via Pierce BCA Protein Assay Kit (Thermo Fisher Scientific, Waltham, MA, USA). Equal amounts of proteins (50 μg/lane) were separated by SDS-PAGE and the resolved proteins were transferred onto nitrocellulose membrane. Transferred membrane was blocked with either 5% skim milk powder or 2.5% bovine serum albumin in Tris Buffered Saline (TBS) with Tween 20 (TBS-T), followed by hybridization with primary and respective secondary antibodies. Antibody-bound protein bands were visualized by Immobilon Western Chemiluminescent HRP substrate (Merck Millipore, Billerica, MA, USA) and exposed to x-ray film. MyImageAnalysis™ Software version 1.1 (Thermo Fisher Scientific) was used for lane profile densitometry and analysis. Anti-PGR antibody clone H190, anti-ERα antibody (MC-20: sc-542) were from Santa Cruz Biotechnology (Dallas, USA). Anti-RANKL antibody (Abnova, PAB12974) was obtained from Abnova (Taipei, Taiwan). Anti-WNT4 antibody (Abcam, AB91226) was obtained from Abcam (Cambridge, United Kingdom).

### Histopathology and immunohistochemistry

Freshly harvested mammary glands were fixed in 4% paraformaldehyde and dehydrated through a series of graded ethanol followed by mixed xylenes and paraffin prior to embedment. Several histological sections of 5 µm thick were prepared from the paraffin-embedded tissues and stained with H&E and/or examined by immunohistochemistry (IHC). For IHC, tissue sections were dewaxed, rehydrated, and treated with citrate buffer (pH 6) at 95 °C for 10 min to unmask the antigen epitopes. Hydrogen peroxidase of 3% was used to block endogenous peroxidase activity. Sections were then incubated with anti-RANKL antibody (AF462 from R&D Systems, Minneapolis, USA) for 2 h at room temperature, biotinylated secondary antibody (1:200) overnight, and subsequently stained with VECTASTAIN Elite ABC HRP Kit (Vector laboratories, Burlingame, CA, USA) to enhance signal sensitivity. Immunoreactive cells were visualized with 3,3’-diaminobenzidine chromogen in brown and the whole sections were counterstained with haematoxylin.

### Statistical analysis

Statistical analysis of animal studies with two experimental groups was evaluated by Student’s t test. A *p* value of less than 0.05 was considered as statistically significant. Numerical data were expressed as mean ± standard error of the mean (SEM).

## Supplementary Information

Supplementary Figure 1.

Supplementary Figure 2.

Supplementary Figure 3.

Supplementary Figure 4.

Supplementary Figure 5.

## Data Availability

The AF1_QQQ and AF1_FFF mice are available from the The Jackson Laboratory. The strain are MMRRC: [67159-JAX], C57BL/6 J-Pgrem1Lvntu/Mmjax and MMRRC: [67160-JAX], C57BL/6 J-Pgrem2Lvntu/Mmjax, respectively. Materials described in the manuscript, including all relevant raw data, will be freely available to any researcher wishing to use them for non-commercial purposes.
