# Supplementary figures and images for: Activation function 1 of progesterone receptor is required for mammary development and regulation of RANKL during pregnancy
Source: Sci Rep. 2022 Jul 19;12:12286. doi: 10.1038/s41598-022-16289-x (PMC9296660; doi:10.1038/s41598-022-16289-x)

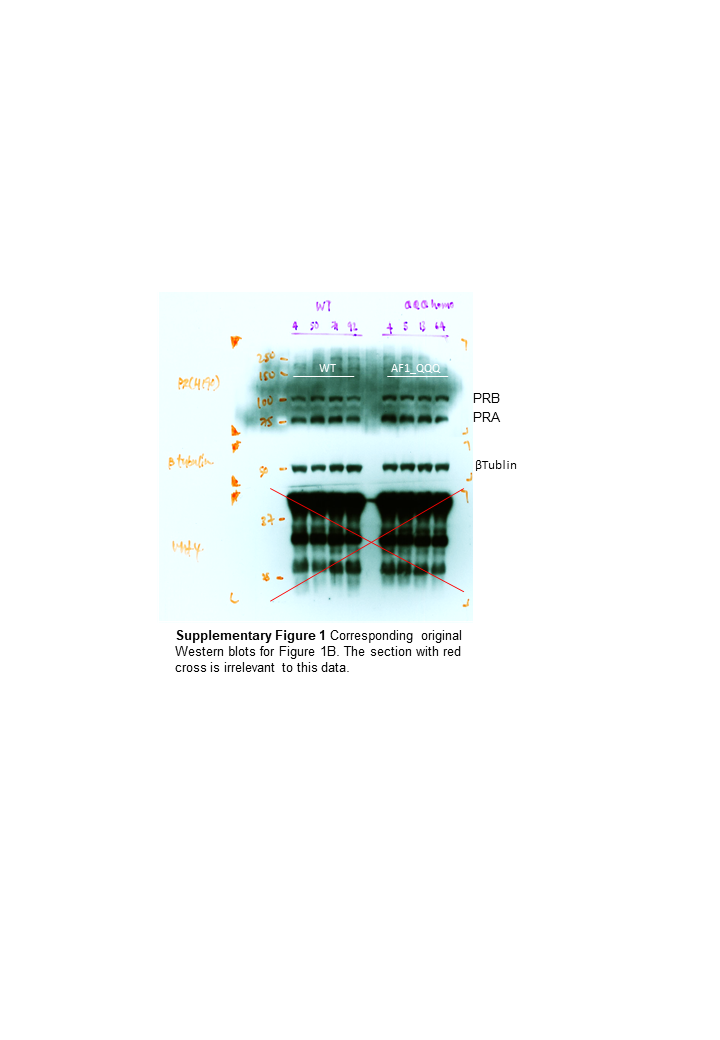

Supplement: Supplementary file 1 — Supplementary Figure 1. [file 41598_2022_16289_MOESM1_ESM.tif]

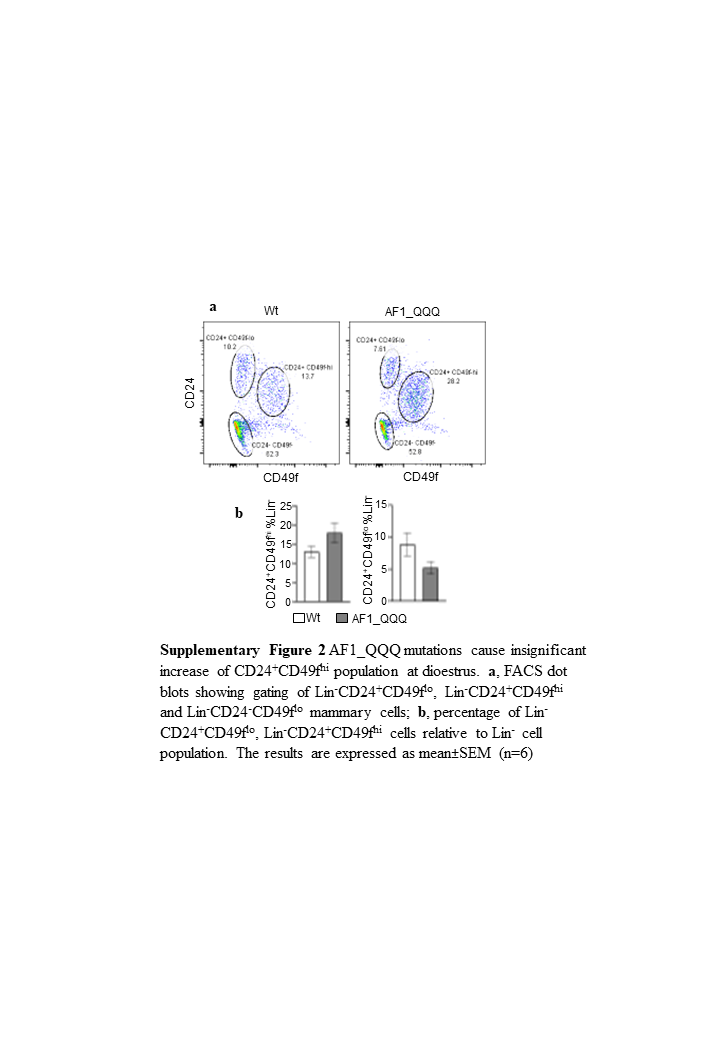

Supplement: Supplementary file 2 — Supplementary Figure 2. [file 41598_2022_16289_MOESM2_ESM.tif]

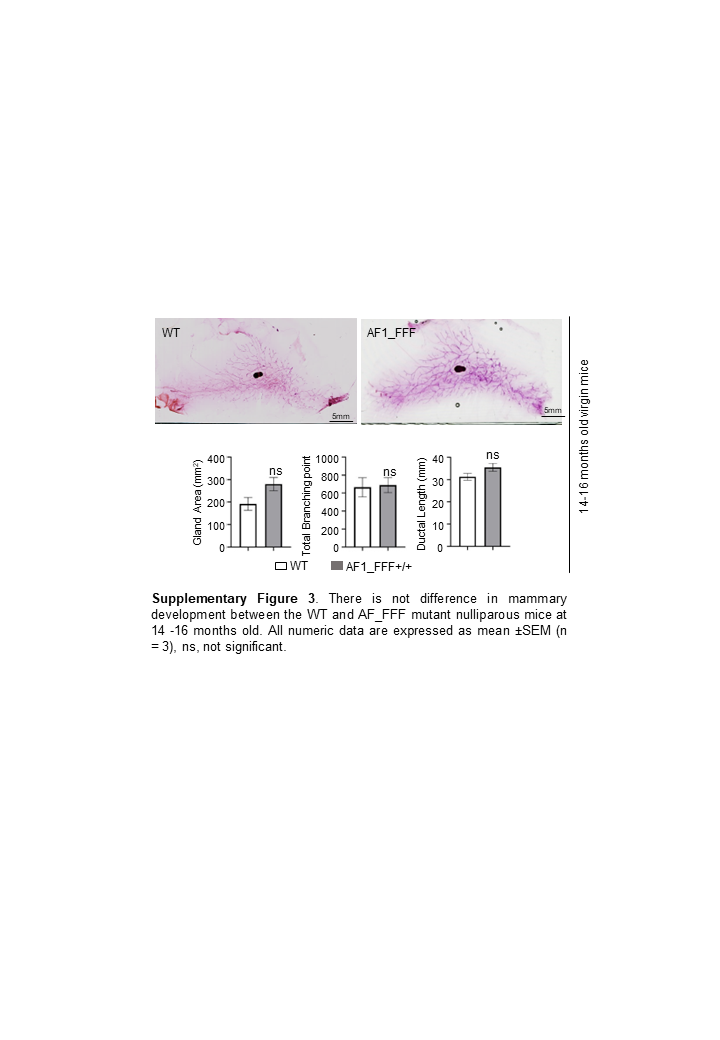

Supplement: Supplementary file 3 — Supplementary Figure 3. [file 41598_2022_16289_MOESM3_ESM.tif]

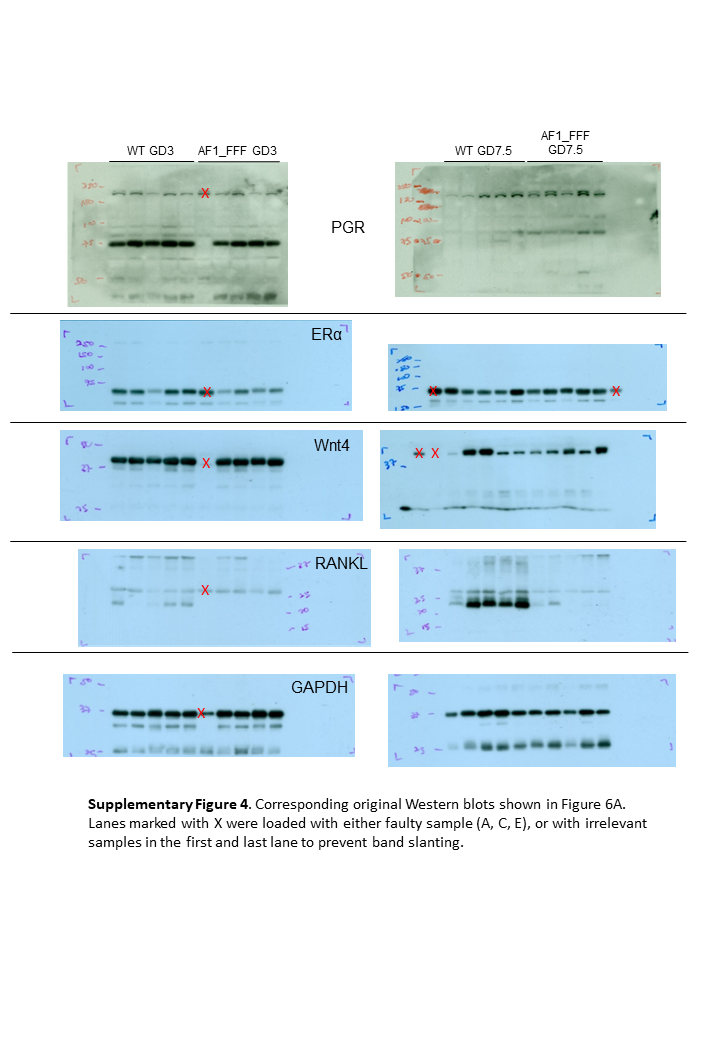

Supplement: Supplementary file 4 — Supplementary Figure 4. [file 41598_2022_16289_MOESM4_ESM.tif]

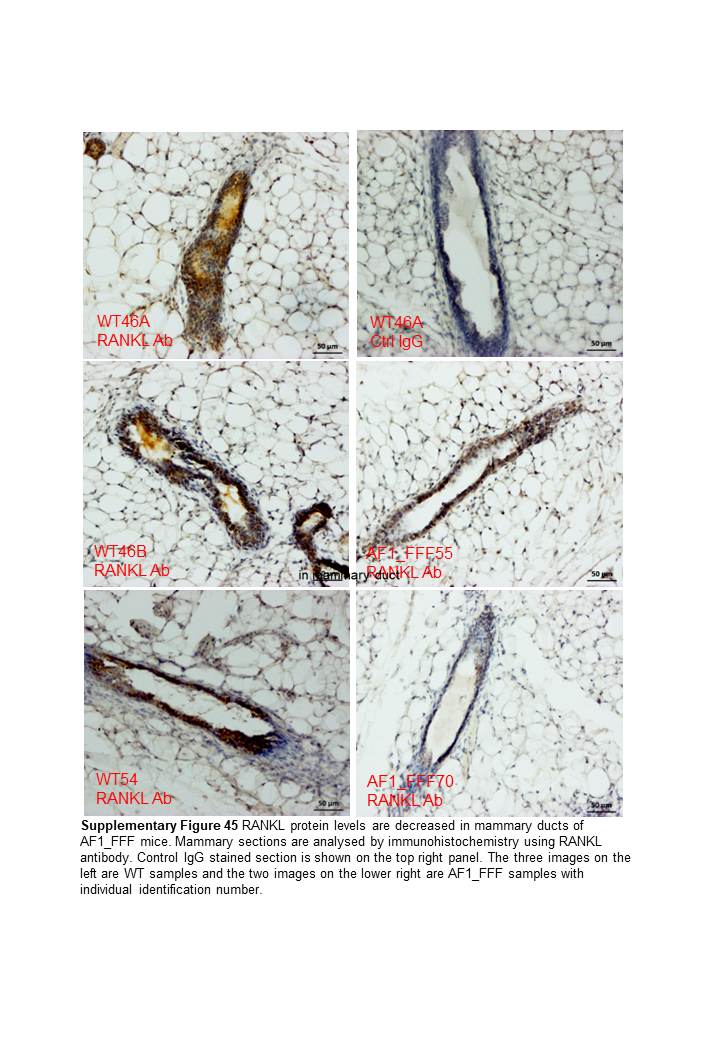

Supplement: Supplementary file 5 — Supplementary Figure 5. [file 41598_2022_16289_MOESM5_ESM.tif]
